# Collagenofibrotic glomerulopathy – Case report with review of literature

**DOI:** 10.4103/0971-4065.78080

**Published:** 2011

**Authors:** K. C. Patro, R. Jha, M. Sahay, G. Swarnalatha

**Affiliations:** 1,2 Department of Nephrology, Medwin Hospital, Hyderabad, India; 2 Department of Nephrology, Osmania Medical College, Hyderabad, India; 3 Department of Pathology, Apollo Hospital, Hyderabad, India

**Keywords:** Collagenofibrotic glomerulopathy, fibrils, asymptomatic proteinuria, fibrillary material

## Abstract

Collagenofibrotic glomerulopathy is a rare, idiopathic glomerular disease characterized by abnormal accumulation of type III collagen fibrils within the mesangial matrix and subendothelial space and a marked increase in serum type III procollagen peptide levels. Proteinuria (commonest feature), edema, hypertension, and occasional progression to end-stage renal disease are the various features of this disease. The etiology and pathogenesis remain elusive. There have been reports of the disease running in the family, suggesting the possibility of genetic transmission. We report two cases of this rare entity.

## Introduction

Collagenofibrotic glomerulopathy, first reported in 1979 by Arakawa *et al*. is characterized by abnormal accumulation of type III collagen fibrils with in the mesangial matrix and subendothelial space and a marked increase in serum type III procollagen peptide levels.[1] It was initially considered to be a variant of nail-patella syndrome with no skeletal deformities, as the glomeruli in nail-patella syndrome too showed accumulation of collagen. In 1990s, this entity was recognized as a separate entity and has since been included into the classification of glomerular diseases.[2] Proteinuria (commonest feature), edema, hypertension, and occasional progression to end-stage renal disease are the various features of this disease. So far, only about 40 cases have been reported in the literature. We report here two cases of this rare entity.

## Case Reports

### Case 1

A 43-year-old perimenopausal female, recently detected hypertensive (since 6 months), nondiabetic and with no other significant past or family history presented to us for evaluation of hypertension. She was on amlodepine 5 mg/day and atenolol 25 mg/day. She had no history of edema. The general and systemic examination was unremarkable except for high blood pressure (190/110 mmHg no asymmetry or postural drop). Fundus was normal. Evaluation revealed normal hemogram (hemoglobin 13.3 g/dl, TLC 10,200/cumm, and normal platelet count and peripheral smear). Proteinuria with microscopic hematuria and bland sediment was noted on urine examination. Proteinuria was quantified to be 5.83 g per day. There was fasting hyperglycemia (FBS – 132 mg/dl), normal serum creatinine (0.84 mg/dl), and lipid profile. Ultrasonography revealed normal size kidneys and no other abnormality. Coagulation profile was normal, virus screen for HIV, HBsAg and anti-HCV was negative; protein electrophoresis and immunofixation showed a normal pattern with no abnormal protein bands.

**Figure 1:**
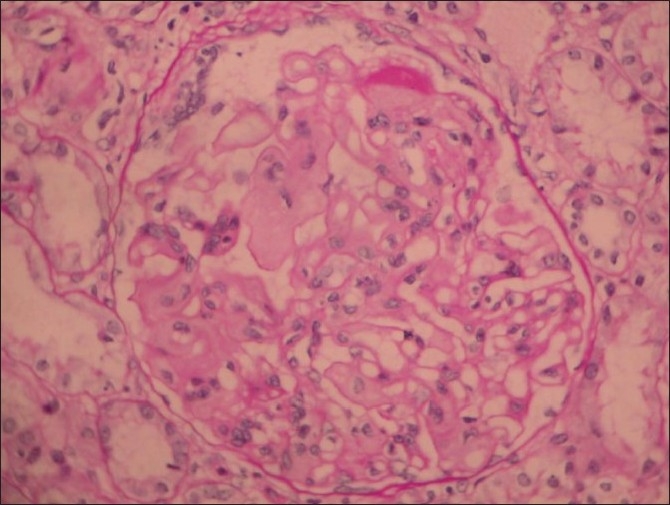
Periodic acid Schiff (PAS) stain, 400× magnification, shows an enlarged glomerulus with deposits which are PAS negative and occasional PAS positive hyaline caps. Tubules and interstitium are normal

**Figure 2:**
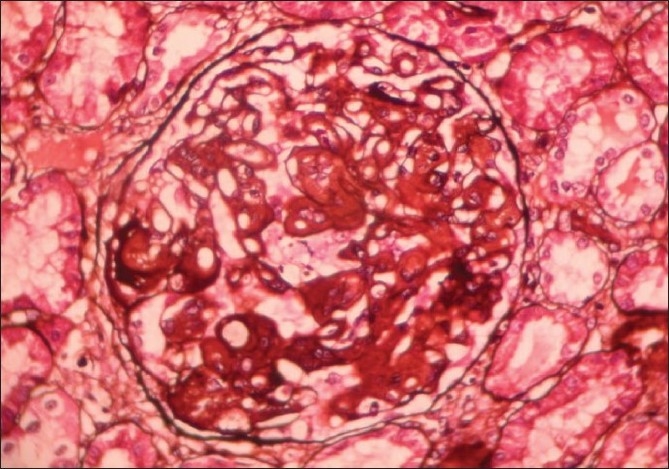
Silver stain, 400× view shows deposits are silver negative

**Figure 3:**
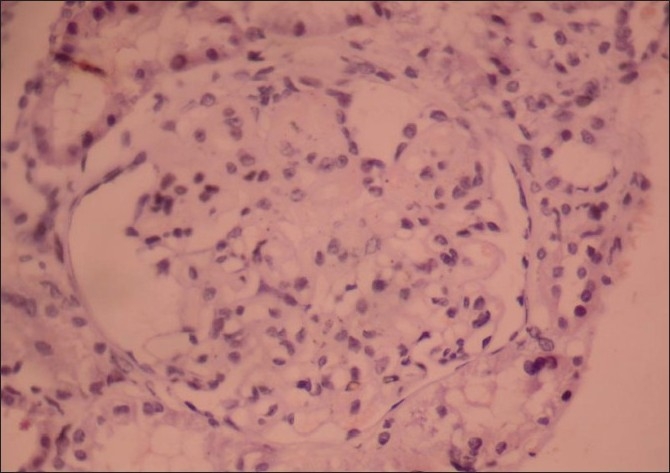
Deposits were not congophilic

**Figure 4:**
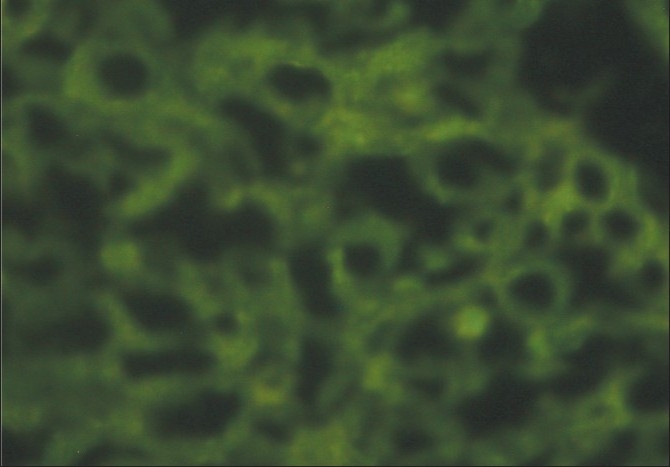
Immuno-fluorescence stain: the deposits were negative for all IgG, IgM and complements

**Figure 5:**
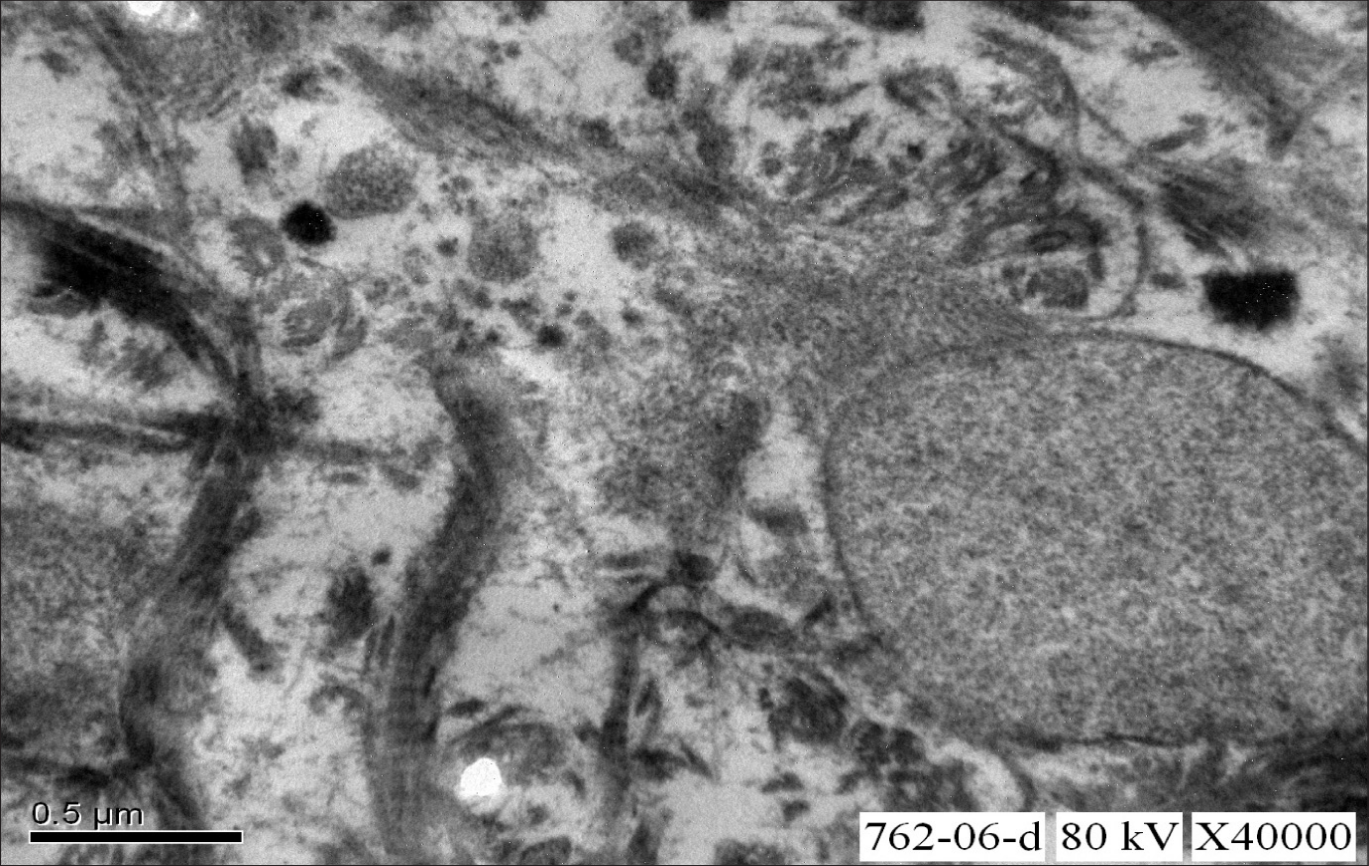
Electron microscopic study of renal biopsy specimen fixed using gluteraldehyde, viewed under 40,000× magnification revealed abundant subendothelial deposits of large fibers (97nm in width) with long spacing striations suggestive of collagen fibers; confirming the diagnosis of collagenous glomerulopathy

A renal biopsy performed showed 14 glomeruli which were enlarged, a prominent mesangial widening with an eosinophilic fibrillary appearance and a peripheral interposition along with basement membrane giving it a thickened appearance. These deposits were negative for periodic acid Schiff (PAS) [Figure 1], silver methanamine [Figure 2], and Congo red stains [Figure 3]. There was associated PAS positive peripheral hyalinosis in some glomeruli [Figure 1]. The glomeruli showed no increase in cellularity, segmental lesions, or crescents. Few tubules had eosinophilic casts within and the interstitium showed a patchy mild lymphomononuclear infiltrates. The larger arteries showed intimal sclerosis. Direct immunofluorescence study showed focal and segmental deposits of C3c corresponding to peripheral hyalinosis [Figure 4]. These features were suggestive of a deposit glomerulopathy – a fibrillary or a collagenous nephropathy. Electron microscopic study was performed to identify conclusively the type of deposits. The biopsy cores fixed with gluteraldehyde revealed abundant subendothelial deposits of large fibers (97 nm in width) with long spacing striations (25–30 nm), which were characteristic of collagen fibers [Figure 5]. These features were suggestive of a definitive diagnosis of collagenous glomerulopathy.

She was initiated on ramipril 10 mg/day and losartan 100 mg/day and gliclazide 60 mg/day. At last follow up (3 years after diagnosis), partial remission of proteinuria was noted (decreased from 5.83 to 2.8 g/day), her blood pressure is controlled (130/80 mmHg) and the renal function has remained stable (serum creatinine – 1.09 mg/dl, eGFR – 75 ml/min).

### Case 2

A 20-year-old male, a known hypertensive presented with pedal edema of 4 years duration. There was no other significant past history of connective disease or family history of renal disease. On examination, patient had a blood pressure of 160/90 mmHg and mild pedal edema. Fundus revealed grade II hypertensive retinopathy. Other general physical examination and systemic examination was normal.

Laboratory results included a hemoglobin level of 8 g/dl, white blood cell count of 11,000/cumm, blood urea of 55 mg/dl, serum creatinine of 2.0 mg/dl, estimated CCR of 50 ml/min, total cholesterol of 224 mg/dl, total serum protein level of 5.6 g/dl, and a serum albumin level of 2.4 g/dl. Urine was having 3+ proteinuria with no RBCs and the 24-h urinary protein was 3.4 g/24 h. Ultrasound scan of abdomen revealed normal sized kidneys (right kidney of size 11.3 cm × 6.0 cm and a left kidney of size 12 cm × 6.2 cm) with grade II increased echotexture and mild hepatomegaly.

**Figure 6:**
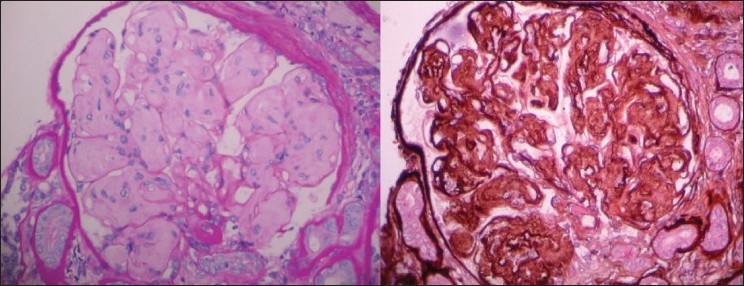
Kidney biopsy slide stained with hemotoxylin-eosin stain and periodic acid Schiff stain (PAS), viewed under 400× magnification shows an enlarged glomerulus with increased mesangial matrix and mesangial interposition with diffuse membrane thickening. Tubules and interstitium are normal

**Figure 7:**
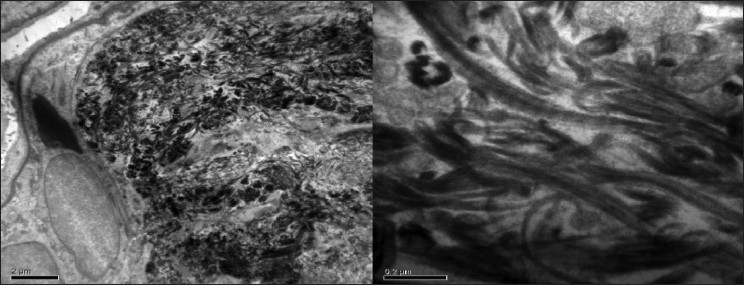
Electron microscopic study of renal biopsy specimen fixed using gluteraldehyde, viewed under 40,000× magnification revealed abundant subendothelial deposits of large fibers (97nm in width) with long spacing striations suggestive of collagen fibers; confirming the diagnosis of collagenous glomerulopathy

The patient underwent renal biopsy. Light microscopy and immunoflourescence revealed features suggestive of collagenofibrotic glomerulopathy [Figure 6], and was confirmed by electron microscopy [Figure 7]. He was stable clinically and biochemically till last 1 year

## Discussion

Collagenofibrotic glomerulopathy, described mostly from Japan, has been recognized as a distinct entity. The etiology and pathogenesis are unknown. It is usually sporadic and when noticed in siblings the entity has been considered to be an autosomal recessive trait. There has been a speculation whether it is a primary renal disease or manifestation of a systemic disease, i.e., the abnormal collagen is from within the glomerulus or from an extrarenal source.[3]

It has been noted that type III collagen, a ubiquitous extracellular structural protein seen only in the interstitium and blood vessels of a normal glomerulus, is also produced by mesangial cells as seen in cell cultures and *in situ* hybridization techniques, explaining a local cause for collagen deposition. Interleukin 4 (IL-4) selectively stimulated type III collagen synthesis in human glomerular cells and IL-4 – neutralizing antibodies have prevented renal disease in IL-4 transgenic mice, which developed glomerulosclerosis in the absence of immunoglobulin deposition. These observations have suggested that the disease is a local pathology. Alternatively, the identification of hepatic perisinusoidal fibrosis in collagenofibrotic glomerulopathy and the finding of abnormally high serum procollagen III N-terminal peptide (PIIINP) levels suggest the systemic nature of this disease.[4]

Clinically, the most common presenting feature is edema and/or persistent proteinuria that may be of nephrotic range (about 60% of patients). Both the cases discussed here had nephrotic proteinuria but only one of them had edema. Hypertension is seen in two-third of cases at the time of presentation. Anemia may be noticed even before the development of renal dysfunction. Occasionally, microangiopathic hemolytic anemia has been documented in children. Extrarenal symptoms of skeletal or nail abnormalities are absent, unlike in cases of nail-patella syndrome. The natural history of the disease is variable, but is one of inexorable progression. Children are more likely to progress to end-stage renal failure. Successful renal transplantation without recurrence has been documented in one case.

Apart from very high PIIINP levels, other laboratory data are nonspecific. We could not estimate procollagen peptide levels or perform immunohistochemistry for collagen typing. Renal function tests are usually normal or slightly increased at presentation. Urine examination occasionally shows microscopic hematuria and proteinuria but no active sediments. Serologic tests for autoimmunity are negative and monoclonal immunoglobulins are absent in serum or urine.

Pathologic findings on light microscopy are suggestive of a deposition disease. Light microscopy reveals globally enlarged glomerular tufts because of the eosinophilic material in the capillary walls and the mesangium. This material is weakly PAS positive, but Congo red and thioflavin T negative. It also shows strong blue staining with periodic acid-methanamine silver, Aniline blue, and Mason trichrome stains. The thickened peripheral capillary walls resemble the double contour appearance of membranoproliferative glomerulonephritis. No adhesions or crescents are noted. In advanced stages, capillary lumens are narrowed by expanded mesangium and thickened capillary walls and glomeruli show a nodular appearance resembling Kimmelstiel–Wilson lesions. Patchy tubular atrophy and interstitial fibrosis may be present. Arteriolar hyalinosis and thickening of walls of arteries are sometimes seen, secondary to hypertension. Immunofluorescence staining is usually negative.

Immunohistochemistry for specific collagen types shows abundant staining for type III collagen. Electron microscopy is imperative to establish a diagnosis so as to differentiate the immunofluorescence negative deposition diseases such as collagenofibrotic glomerulonephritis, fibronectin glomerulopathy and diabetic glomerulosclerosis. Electron microscopy identifies marked accumulation of fibrillary material in mesangium and subendothelial space of glomerular basement membrane. The material is identified easily on routine staining (gluteraldehyde) or special staining as tannic acid–lead or phosphotungstic acid may be used. The fibers have a transverse band structure with distinct periodicity of approximately 60 nm, same as seen in type III collagen. The fibers are usually curved or frayed (unlike the fibers in the interstitium which are usually straight), forming irregularly arranged bundles on longitudinal section and flower-like or ragged moth-eaten appearance on cross-section. The microtubules as seen in immunotactoid glomerulopathy, fibrin, and amyloid fibrils are not seen. In contrast to nail-patella syndrome, the lamina densa of the glomerular basement membrane in collagenofibrotic glomerulopathy is of normal thickness and lacks the lucent areas or the so-called moth-eaten appearance.

No specific therapy is available. Supportive measures to control hypertension and diuretics to relieve edema are to be employed. Dialysis therapy or renal transplantation may be required for patients who reach end-stage renal disease. Steroid use has been anecdotal and there is no conclusive evidence to prove its efficacy in this disease.

Both cases discussed in this paper were surprise diagnosis on histopathology and suggest the need for electron microscopy for confirmation of this entity. Relative stability of renal dysfunction in both the cases in short term (3–5 years) follow up (from the beginning of symptoms) with standard care therapy looks reassuring. To the best of our knowledge, this could be the first reported case study from India.

To conclude, any type of deposition disease on light microscopy, either immunofluorescence positive or negative should be subjected to electron microscopy so as to differentiate the various fibers based on the size, shape, or presence of transverse bands and make a confirmatory diagnosis.
